# Bridging Molecular Insights and Agronomic Innovations: Cutting-Edge Strategies for Overcoming Boron Deficiency in Sustainable Rapeseed Cultivation

**DOI:** 10.3390/plants14070995

**Published:** 2025-03-21

**Authors:** Muhammad Riaz, Muhammad Rafiq, Hafiz Husnain Nawaz, Weiguo Miao

**Affiliations:** 1 Guangdong Engineering and Technology Center for Environmental Pollution Prevention and Control in Agricultural Producing Areas, College of Resources and Environment, Zhongkai University of Agriculture and Engineering, Guangzhou 510225, China; riaz1480@hotmail.com; 2 Jiangxi Key Laboratory for Sustainable Utilization of Chinese Materia Medica Resources, Lushan Botanical Garden, Chinese Academy of Sciences, Jiujiang 332900, China; rafiqsabqi@gmail.com; 3 Lushan Xinglin Institute for Medicinal Plants, Jiujiang Xinglin Key Laboratory for Traditional Chinese Medicines, Jiujiang 332900, China; 4 Faculty of Agricultural, Environmental and Food Sciences, Free University of Bozen Bolzano, 39100 Bozen-Bolzano, Italy; hnawaz@unibz.it; 5 Key Laboratory of Green Prevention and Control of Tropical Plant Disease and Pests, Ministry of Education, College of Plant Protection, Hainan University, Haikou 570228, China; 6 Danzhou Invasive Species Observation and Research Station of Hainan Province, Hainan University, Dazhou 571737, China

**Keywords:** boron, rapeseed, transporters, NIPs, BORs

## Abstract

Boron (B) is an essential micronutrient for the growth, development, and maintenance of cellular integrity in vascular plants, and is especially important in cell wall synthesis and reproductive development. Rapeseed (*Brassica napus* L.), one of the dominant oil crops globally, has a high boron demand and its yield is dramatically decreased under B-deficiency conditions. Rapeseed, which is very sensitive to boron deficiency, suffers from reduced growth and reproductive development, ultimately causing severe yield losses. Here, we reviewed the present state of knowledge on the physiological function of boron in rapeseed, mechanisms of boron uptake and transport, specific effects of boron deficiency in rapeseed, and approaches to alleviate boron deficiency in rapeseed at the agronomical and molecular levels. A specific focus is given to recent molecular breakthroughs and agronomic approaches that may improve boron efficiency. The review focuses on practices that may alleviate the problems caused by boron-deficient soils by investigating the genetic and physiological mechanisms of boron tolerance. In summary, this review describes the integration of molecular information with practical agronomy as an important aspect of breeding future nutrient-efficient rapeseed cultivars that can sustain increasing yields while being cultivated in regions with boron-deficient soils.

## 1. Introduction

Boron (B) is a crucial micronutrient that significantly influences plant growth and development, impacting various physiological and biochemical processes both directly and indirectly [1,2]. Most of it is taken up as boric acid, and it is necessary for the stabilization of molecules that contain cis-diol moieties [3]. These groups are essential for maintaining the structural integrity and biological functions of cell membranes and walls [4]. Its principal role is in the assembly and stabilization of the primary cell wall via the interlinking of the pectin polysaccharide rhamnogalacturonan II (RG-II) [3,4,5]. In soil, boron primarily occurs as boric acid (H_3_BO_3_), which has the potential to leach when rainfall is high [1]. Approximately 90% of boron is found in its cell wall [6]. Moreover, boron influences the dynamics of other nutrients such as calcium, nitrogen, phosphorus, potassium, and zinc, which are crucial for plant responses to environmental changes [2]. The margin between boron deficiency and toxicity is narrow, and both hinder plant growth and productivity [1,7]. Boron deficiency can impair the growth of leaves, roots, and flowers, whereas excessive boron may lower photosynthetic efficiency and reduce pollen viability [1,8]. Sustainable agricultural practices are heavily dependent on the development of plant varieties that not only utilize boron efficiently but also tolerate fluctuations in boron levels [1,8].

Low levels of boron can have a pronounced negative impact on many crops grown globally. Over the past 60 years, it has been reported as a limiting factor for 132 distinct crop varieties in more than 80 countries [9]. According to the USDA’s Rapeseed Explorer, which reports 87.103 million metric tons of global rapeseed production, this has resulted in a rapeseed oil yield of 31.8 million metric tons. This global issue focuses on the importance of boron as a micronutrient required for proper plant growth and development. *Brassica napus* is the third most cultivated oilseed crop in the world and an allotetraploid species whose yield is highly dependent on the supply of boron. The term “critical concentration” refers to the lowest level of a nutrient found in a specific part of a plant that is necessary to achieve 90% of the maximum biomass or yield for that particular plant species [10]. The boron needs of plants are typically influenced by two primary factors: the specific species of the plant and its developmental stage [11]. Among various plant species, oilseed crops exhibit the greatest demand for boron, with their requirements during the reproductive phase surpassing those during the vegetative growth stages. The authors of [12] proposed that the increased boron demand during the reproductive growth phase may be attributed to reduced transpiration rates during this period. This decrease in transpiration limits the supply of boron to reproductive structures, which are typically situated at the extremities of plants. Various research efforts have determined the critical B concentration for oilseed rape, but the results show significant variation. These findings are often specific to particular factors such as the timing of sample collection, the plant component analyzed, and the portion of B examined in the plant tissues. As reported by [13], the critical threshold for boron deficiency is identified as 10–14 mg of B per kg of dry matter in the newest fully opened leaf, while the youngest fully developed leaves require a concentration of 6–8 mg of B per kg of dry matter. Conversely, [14] determined that for *B. napus* cultivated in calcareous soils, the critical B concentration was 32 mg kg^−1^ in the entire shoot and 38 mg kg^−1^ in fully developed leaves. The authors of [15] noted characteristic boron deficiency indicators on the foliage of *B. napus* cultivated in nutrient solutions with boron concentrations below 0.3 µM. In a related study, [16] employed a chelating resin to maintain a steady B concentration in the nutrient solution. Their findings revealed that plants experienced a drastic reduction in biomass when the B concentration in the nutrient solution was 0.3 µM or lower. For oilseed rape to reach its highest yield potential, the soil must contain more than 0.5 mg B kg^−1^. However, when the soil boron level reaches 5 mg kg^−1^, signs of B toxicity become apparent [9]. When soil contains insufficient boron (≤0.5 mg B/kg soil), plants may initially appear unaffected. However, this deficiency can result in the production of flowers that fail to develop seeds [17]. Boron deficiency is a common problem in many crops worldwide [9]. In China, boron deficiency has negatively impacted over 33 million hectares of farmland, significantly affecting the growth and productivity of *B. napus*, which is one of the most sensitive crops to low boron availability [18,19].

The oilseed crop *Brassica napus* L. (rapeseed) serves as a vital agricultural commodity throughout the world [20]. Although boron fertilization is of high importance, there is little risk of toxicity, even at high application rates, making boron an effective choice for rapeseed–rice cropping systems [21]. Research on the rapeseed genome, including whole-genome sequencing and molecular markers, has advanced, leading to improvements in seed quality and oil content [20]. Nevertheless, the rapeseed industry continues to face challenges, such as high labor costs and reliance on imports to meet domestic demand, highlighting the need to improve both production efficiency and supply chain resilience [22,23,24]. The multifunctional uses of rapeseed—such as in edible oil production, biodiesel, animal feed, and pollution remediation—further enhance its economic utility and commercial benefits. However, its high sensitivity to micronutrient imbalances, especially boron, has posed challenges for consistent crop production. Boron deficiency can result in reduced cell wall stability, impaired meristem development, and decreased seed quality, making the understanding of its underlying mechanisms and management strategies critical [25]. The rationale for this review is to consolidate current findings on the role of boron in rapeseed physiology, elucidate the mechanisms behind boron uptake and deficiency responses, and review management practices aimed at overcoming boron limitations. This review is intended to guide both researchers and practitioners in developing strategies to improve rapeseed performance under boron stress.

## 2. Boron Deficiency in Rapeseed

**Figure 1:**
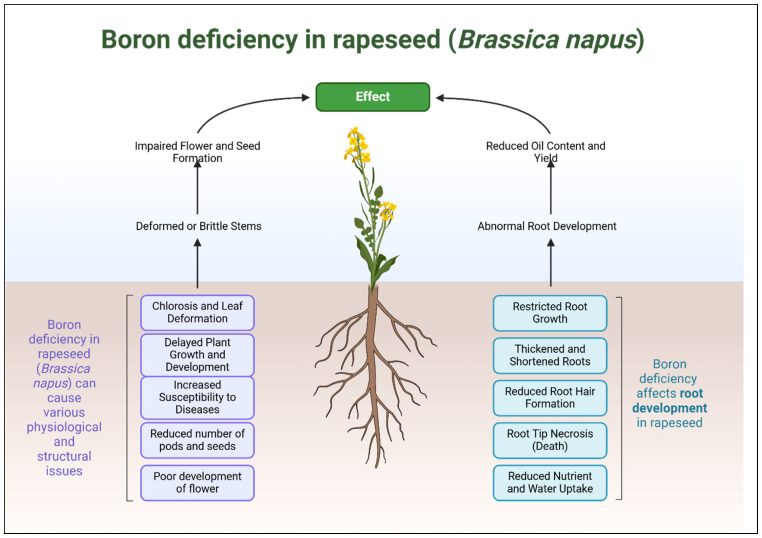
Effects of boron deficiency on rapeseed (*Brassica napus*), highlighting key symptoms such as stunted root growth, thickened and brittle stems, poor flower and seed formation, leaf deformation with chlorosis, reduced pod and seed production, and decreased oil content. Severe boron deficiency leads to necrosis of root tips, structural weaknesses, and increased susceptibility to diseases, ultimately reducing plant vigor and yield.

Low concentrations of boron lead to reduced plant height, shorter taproots, and lower dry weights of roots and shoots, all of which are characteristic of stunted growth (Figure 1). In *Brassica napus*, these deficiencies result in problems such as poor root development, early flower abscission, and reduced seed yields [26]. A lack of boron has detrimental effects on various processes and the mechanical integrity of cell walls, causing cell distortion and increased sensitivity to rupture, particularly in sensitive genotypes [27]. Various *Brassica napus* genotypes exhibit different degrees of tolerance to boron deficiency, with some genotypes showing relatively better cell wall integrity and enzyme activity [27,28]. There is significant variation in growth rates under boron limitation across *B. napus* varieties [17]. Three boron-efficient accessions were chosen through automated high-throughput phenotyping facility screening of 234 spring and 356 winter accessions from Europe and Asia [29]. The genotypes possessing more efficient boron utilization demonstrated larger biomass production together with expanded root systems, resulting in greater seed yields compared to the less efficient genotypes that kept their boron levels much lower than needed [29] (Table 1).

## 3. Mechanisms of Boron Uptake and Transport

### 3.1. Specialized Transport Proteins in Boron Transport

Plants primarily obtain boron from the soil solution in the form of boric acid (H_3_BO_3_). This small, uncharged molecule is absorbed by plant roots directly from the soil, serving as the main source of boron for plant uptake [11]. As an uncharged molecule, boric acid is the predominant chemical form of B accumulated by plants [11]. Approximately 96% of boron found in both soil and plants exists as boric acid, while only a minor fraction is present in the form of the borate anion (B(OH)_4_^−^) [41]. Boron is unique among essential nutrients because plants absorb it as a neutral compound. In acidic soils, the proportion of boric acid increases relative to the borate anion, which results in a higher absorption rate of boron compared to neutral or alkaline soils. Once boron is absorbed by the roots, it is quickly transported to the shoots and reproductive organs via the transpiration stream through the plant’s vascular system [42].

**Figure 2:**
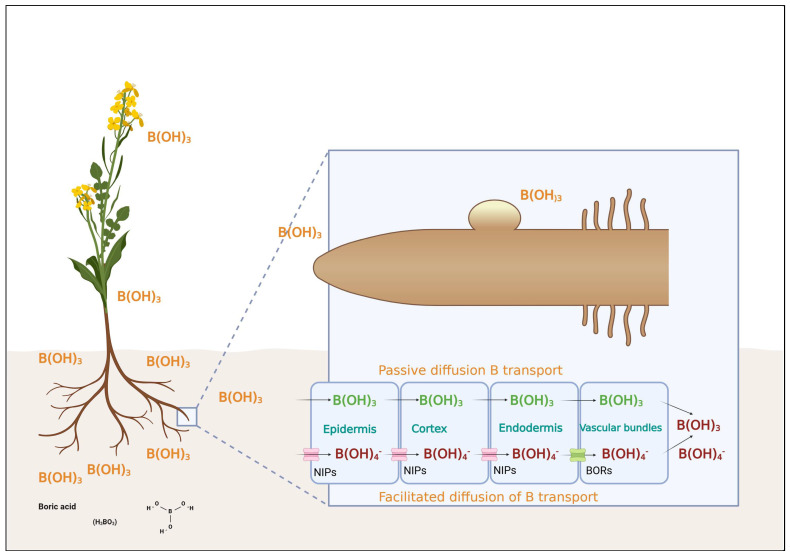
Boron uptake and transport in plants through facilitated and passive mechanisms. Boron is primarily absorbed as boric acid via passive diffusion in the roots, driven by transpiration flow. In conditions of low boron availability, active transport mechanisms involving boron transporters facilitate its uptake. Once inside the plant, boron moves through the xylem to areas of high demand, supporting critical functions such as cell wall integrity, reproductive development, and nutrient transport (adapted from [49]).

The uptake of boron into plant cells of higher plants occurs through both passive diffusion and facilitated or active transport (Figure 2): (1) Passive transport: In soils with high B availability, boric acid (H_3_BO_3_) can enter root cells of higher plants via passive diffusion through lipid membranes or facilitated transport through aquaporins, such as NIP (nodulin 26-like intrinsic proteins) channels. (2) Active transport: At low B concentrations, specialized transport proteins mediate uptake. NIP channels facilitate passive boric acid influx, while BOR1-like transporters actively export boron (as borate, B(OH)_4_^−^) into the xylem, enabling its distribution to growing tissues [43,44,45]. The coordination of these transporters ensures that boron is efficiently allocated to areas of high metabolic demand, particularly in young and developing tissues. There are primary pathways for boron absorption in plants, as illustrated in Figure 2. Under conditions of sufficient boron availability, the passive diffusion of boric acid acts as the predominant mechanism for plasma membrane intake. The absorption of B occurs through passive transmembrane diffusion while being affected by three primary elements: the soil solution boric acid level, transpiration rate, and plasma membrane permeability [25]. Recent studies have described a number of boric acid channels involved in boron uptake under conditions of limited availability. One such channel, NIP5;1, mediates boron influx into *Arabidopsis* root cells [45]. Takano et al. [45] identified NIP6;1 as the third transport route controlling B long-distance translocation between the xylem and phloem and its distribution in the vascular system of the shoot. When B becomes restricted, [6] demonstrated that borate is taken up by plants and enters their xylem via the BOR1 exporter. BOR1 substantially localizes to root pericycle cells to mediate the transport of borate to the xylem structure, despite its natural concentration gradient [6,43]. Transporters including BOR1 and BOR4 actively extrude borate anions from cells, fulfilling crucial roles in xylem boron loading for long-distance transport from roots to shoots and for the prevention of toxic accumulation [43,44,45]. These boron transporters are tightly regulated by boron availability. When boron concentrations are high, the transporters NIP5;1 and BOR1 are downregulated via mechanisms such as mRNA degradation and proteolysis [43,44,45]. Moreover, the localization of these transporters in the cell is conducive to efficient uptake and distribution of boron. In *Arabidopsis*, for instance, NIP5;1 is oriented toward the soil, whereas the heterocomplex BOR1 is directed toward the stele, maximizing boron influx from the soil to plant [43,46]. Boron transporters are conserved across species, with similar transporters identified in both *B. napus* and *Oryza sativa*, suggesting an evolutionary adaptation for boron homeostasis [44,47] (Table 1). The boron-efficient Chinese genotype Qingyou 10 (QY10) in boron-deficient environments showed enhanced growth and seed yield, along with a reduced number of boron-binding sites within cell walls and an improved ability to uptake and transport boron compared to less efficient genotypes [17,19]. Genotype Westar 10 (W10), although boron-efficient in high-boron conditions, demonstrates boron-inefficient traits under boron-limited conditions by accumulating greater proportions of RG-II monomers, which generate more boron-binding sites in the cell walls, leading to increased boron requirements for optimal growth and development [28]. Moreover, a higher amount of BOR2 was detected in QY10 than in W10 under B deficiency [27]. A similar pattern was observed with the BOR2 transporter in *Arabidopsis* [46,48], which is critical for enabling effective RG-II cross-linking in the cell wall. The assay for RG-II dimerization showed that at low boron concentrations, the boron-efficient QY10 produced far more borate-crosslinked RG-II dimers than the boron-inefficient W10 [27]. These results indicate that the greater boron tolerance of *B. napus* can be attributed to both a reduced number of boron-binding sites in the cell wall and more rapid RG-II dimerization rates [27].

The initial evidence linking boric acid channels to boron efficiency in *B. napus* emerged from the discovery of a significant quantitative trait locus related to boron efficiency in a hybrid between the boron-efficient genotype QY10 and the boron-inefficient genotype W10. Analyzing gene expressions together with fine mapping revealed *BnaA03.6* genotypes and *NIP5;1b* to be potential candidate genes located on this QTL [19,28]. NIP5;1b in the root cap plasma membrane allows the effective absorption of boron from soil [50]. *BnaA03.NIP5;1b* exhibited variations between QY10 and W10 genotypes but the expression of this gene varied substantially between these varieties. In conditions where boron was limited, the expression of *BnaA03.ROOT2* and *NIP5;1b* were considerably greater in the roots and leaves of QY10 compared to W10 [51]. QY10 is expressed at higher levels than W10 because it contains one set of CTTTC repeats in its 5′ UTR. Additional genetic studies indicate that the elevated expression of the *BnaA03* gene, along with enhanced tolerance to low boron levels and improved seed yield across various genotypes, is linked to the QY10 allele (*NIP5;1b*) [50]. New marker-assisted breeding initiatives now have potential pathways to develop genotypes that demonstrate tolerance against low boron levels.

Close homologs of *AtNIP6;1*, such as *BnaA02NIP6;1a*, are known to transport boric acid in yeast and oocyte systems [52]. Research indicates that *BnaA02NIP6;1a* shows different expression patterns from *AtNIP6;1* because it exists in various *B. napus* tissues [52,53]. The inability of boron to effectively move to develop inflorescences was particularly clear in flower buds of lines that had RNA interference directed against *BnaA02NIP6;1a* [53]. Symptoms of boron deficiency were most obvious in plant lines with RNA interference targeted at *BnaA02NIP6;1a*, suggesting that it is a key *BnaNIP* involved in importing boron into the developing inflorescence [53]. Under boron scarcity, proteins belonging to the NIP-I and NIP-II subgroups, including NIP3, NIP4, NIP5, NIP6, and NIP7, are of particular interest because they are highly expressed in different reproductive structures such as flowers, rachises, peduncles, and pedicels in a European winter genotype [52]. NIP characterization in *Xenopus oocytes* showed that NIP2, NIP3, and NIP4 were also capable of transporting boric acid, in addition to the previously identified boric acid transporters NIP5, NIP6, and NIP7 [52]. The functional manifestation of *Arabidopsis* mutant *Atnip5;1* was demonstrated through promoter regulation of *AtNIP5;1* [52]. These newly discovered boron-transporting members of the NIP-I family of proteins (NIP2, NIP3, and NIP4) are important for maintaining boron homeostasis.

### 3.2. Aquaporins in Boron Transport

Aquaporin proteins are also known to contribute to the regulation of boron transfer in the plant system, thereby facilitating adequate boron homeostasis and enhancing plant growth. Specifically, aquaporins in the nodulin-26-like intrinsic protein (NIP) subgroup promote the transport of boron across the plant cell membrane. These proteins facilitate boron diffusion and are essential for plant growth and development [47,51,54,55,56]. Aquaporins are integral in regulating the uptake and distribution of boron in plant tissues and thus help maintain boron homeostasis. This homeostasis is essential, as both the under- and over-accumulation of B can harm the overall growth of plants [47,54,57].

Under boron-deficient conditions, certain aquaporins, including those in *B. napus*, exhibit altered expression patterns in response to boron stress. During periods of low boron availability, NIPs are upregulated to enhance boron uptake [51]. The aquaporin protein tassel-less1 (TLS1) plays an indispensable role in boron transport, having pronounced effects on vegetative and reproductive growth in maize. Since *TLS1* mutants exhibit phenotypes characteristic of boron deficiency, the gene appears to play an important role in transport and plant development [57]. Moreover, aquaporins such as *BvCOLD1* in sugar beet transport boron and increase tolerance to multiple abiotic stressors, including boron deficiency [58]. Therefore, these proteins are indispensable for boron transport in plants, which is beneficial for maintaining boron homeostasis to support growth under diverse environmental conditions. Their function in transporting boron and other small molecules highlights their significance in plant physiology and physiological responses under stress.

### 3.3. Genetic and Molecular Insights

Numerous quantitative trait loci (QTL) associated with B efficiency have been associated with rapeseed based on genetic analyses. Large and small loci controlling yield-related traits in low B conditions have been shown [59,60]. Candidate genes have been identified from recent fine-mapping and digital gene expression analyses including, but not limited to, *BnaA3* nod26-like intrinsic protein NIP5;1 and *BnaC4.BOR1;1c*, a homolog of an efflux transporter essential for B translocation [61,62]. Genetic studies have found that there are differences in B efficiency among different cultivars of *B. napus*, and some of them can positively respond to boron deficiency [19,29,63]. Important genes and transcription factors, especially *BnaC4.BOR2* and *BnaA9*, have been identified as key for both boron uptake and the ability to withstand boron deficiency [64,65] (Table 2). Several large-effect QTL associated with boron efficiency have been mapped, which can be targeted for plant breeding [19,59]. *BnaC4* is a BOR transporter of the BOR subgroup. *BOR1;1c* and *BnaC4.BOR2* play a crucial role in the absorption and translocation of boron from roots to aerial parts, supplying it to growing tissues, such as leaves and flowers, especially under boron-deficient conditions [36,52,64,66].

BOR transporters are regulated according to boron availability. The expression of *BnaC4* increases when boron availability is low, enhancing the acquisition and distribution of boron [36,62,66]. Several different BOR transporters show tissue-specific expression. Specifically, *BnaC4.BOR1;1c* is mainly expressed in vascular tissues, where it regulates boron movement from roots to shoots and within shoots [36,62]. This tissue-specific expression is important for alleviating boron deficiency in sensitive organs, such as flower buds [52,62]. In *Arabidopsis*, the BOR transporter family comprises functionally diverse genes responsible for the widespread absorption of boron and adaptation to varying boron environments [44,66,79], resulting in a homeostatic mechanism in which the *B. napus* BOR transporter family members have diversified. *B. napus* contains six BOR1 homologs [66], which determine their varying expression patterns throughout their yearly life cycle [52,66]. *BnaC04* mRNA levels displayed high similarity to *AtBOR1* expression, yet maintained extremely low boron-inducible activity in inflorescences from QY10, since this line exhibited B efficiency. When subjected to boron-limiting conditions, *BnaC04.BOR1;1c* displayed elevated expression levels in the European winter genotype compared to other genotypes [52].

The authors of [52] explored novel YABBY protein-encoding genes, such as *BnaA05.BOR1;1c* and *BnaA03*. Their qRT-PCR analysis revealed that *BOR1;3a* was activated in QY10 under conditions of boron deficiency, whereas no such induction was observed in Darmor-PBY018 [52]. An investigation with GFP fusion demonstrated that FN3-*BnaC04.BOR1;1c* exists in the plasma membrane [62]. Depletion of *BnaC04* or the insertion of *BOR1;1c* into the boron-efficient root-specific QY10 genotype resulted in decreased levels of boron in flower organs, causing drooping of flower organs and reduced yield. The expression of *BOR1;1c* led to shorter roots and taller shoots than non-transgenic W10 plants, thus maintaining plant vitality [66]. Like *AtNIP5;1*, *BnaC04.BOR1;1c* is regulated in a 5′-UTR-dependent manner [36]. *BnaC04* localizes at the pole while *BOR1;1c* positions on the stele side of the plasma membrane within root vascular cells. This arrangement is likely advantageous for effective xylem loading [36]. Additionally, the localization of *BnaC04.BOR1;1c* in the cambium cells of shoot nodes towards the phloem was consistent with its role in xylem–phloem transfer [36]. In conclusion, BOR transporters in *B. napus* species play a vital role in modulating B intake and distribution dynamics, particularly in the context of deficiency. They facilitate the effective translocation of boron to the most critical tissues, where it supports plant growth and reproductive success [80]. These transporters thus represent an evolutionary adaptation of *Brassica* for osmoregulation, with varied expressions further corroborating this function.

### 3.4. Transporter Families and Mechanisms of Boron Deficiency Tolerance in Brassica Species

Two key families of proteins responsible for B acquisition and distribution in plants are NOD26-INV(X) (NF) and BOR transporters. NIPs mediate boric acid diffusion across lipid bilayer membranes, while BOR transporters export borate from the cells. Notably, some isoforms of NIPs and BORs, such as NIP5, NIP6, NIP7, and BOR1, are major boron transporters in *B. napus* under boron-deficient conditions [44,52]. *BnaC4.BOR1;1c* and *BnaC4.BOR2* are critical BOR transporters required for boron delivery to reproductive organs and are essential for plant fertility. Additionally, [36,62,64] have reported that BOR2, as a boron transporter, regulates the transport and uptake of boron, especially in low-boron availability environments.

The mRNA levels of *BOR1;1c* are reduced in the presence of boron, demonstrating a negative correlation between mRNA expression levels and boron availability. The regulatory machinery governing this process is essential for efficient boron acquisition and its allocation to sink tissues and developing organs [36]. Transcription factors such as *BnaA9.WRKY47* induce a boron-responsive gene set, including the boron transporter *BnaA3.NIP5;1*, which promotes boron uptake [65]. WRKY transcription factors have been implicated in boron efficiency [65]. Both *BnaNIP5;1s* and *BnaBOR1s* contain multiple W boxes (T/CTGACC/T) in their respective promoter regions, which are known binding sites for WRKY proteins. Yeast one-hybrid (Y1H) assays showed that the W boxes in boron-responsive *BnaNIP5;1s* and *BnaBOR1s* bind *BnaWRKYs* [65]. The mRNA levels of *BnaA03* were higher in plants overexpressing *BnaA9*. Four WRKY47 homologs were markedly upregulated, and *MRN41* was downregulated in its MAR mutants, while other *BnaNIP5;1s* and *BnaBOR1s* showed little fluctuation in transcript levels. Additionally, the *BnaA9* gene was purified from CRISPR/Cas9-mediated mutants in *B. napus*. At low-boron levels, WRKY47 displayed no symptoms of boron deficiency, while lines overexpressing *BnaA9* showed that WRKY47 overexpression conferred lower sensitivity to low-boron stress compared to wild-type plants. The correlation analysis suggested that *BnaWRKYs* is crucial in how *B. napus* responds to a lack of B [65].

The gene *BnaA3.NIP5;1* encodes a boric acid channel that transports boron into root tips. Interestingly, variants of this gene, especially a CTTTC tandem repeat located in its 5′ UTR, correlate with enhanced growth and seed yield under boron-deficient conditions in plant cultivation [50]. Moreover, the gene *BnaC4* and the expression of BOR2 specifically in roots (as opposed to shoots) are important for the plant’s ability to adapt to low boron availability [64], as is the ability to both uptake and translocate boron. In addition, *BnaA9* is a transcription factor, and *BnaA3* is upregulated by WRKY47 in response to low-boron environments, enhancing boron uptake efficiency through *NIP5;1* [65]. The pectin content has been implicated in the mechanical properties of cell walls, which correlate with varying tolerance to low boron levels among rapeseed genotypes. The genotype ‘QY10’ has stronger cell walls and is more tolerant to boron deficiency than the genotype ‘W10’, which possesses porous cell walls [81]. Boron deficiency also reduces the levels of phytohormones, such as indole-3-acetic acid, while increasing those of jasmonates and abscisic acid. Lower-boron genotypes experience more pronounced phytohormone balance disruptions under low-boron conditions [81].

**Figure 3:**
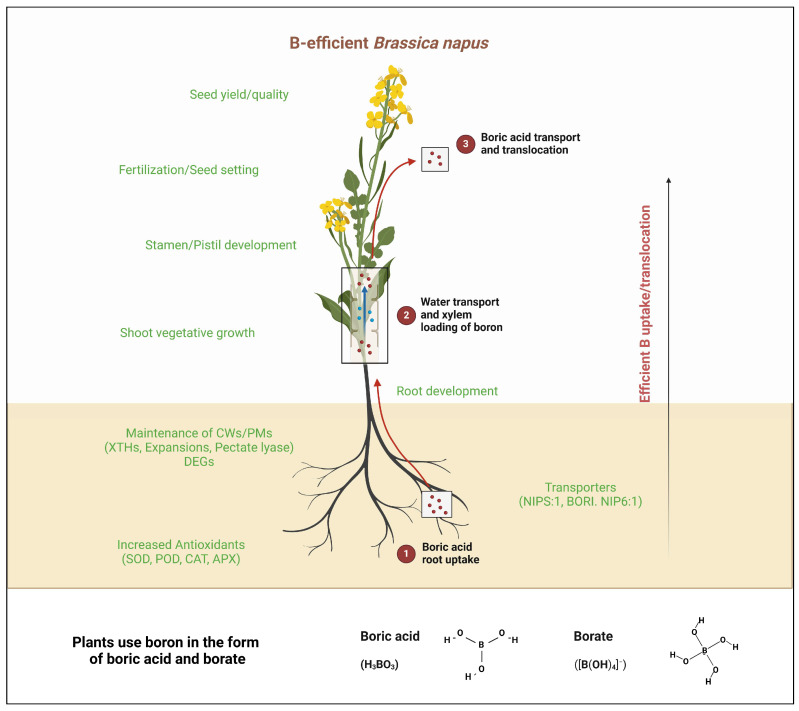
Proposed model explaining the differential responses to B deficiency in efficient rapeseed genotypes. Efficient genotypes exhibit enhanced boron uptake, maintain better root growth, and adapt by optimizing internal boron transport to critical tissues. These genotypes show improved flower and seed formation, reduced structural damage, and maintained higher yield under boron-deficient conditions.

Screening techniques with increased throughput have identified cultivars that appear boron-deficiency-tolerant, including CR2267, CR2280, and CR2285, which exhibit increased boron utilization efficiency and distinct growth patterns compared to others [29] (Figure 3). Several loci associated with boron efficiency have been mapped through quantitative trait locus analysis, establishing a genetic basis for the selection of boron-efficient cultivars using marker-assisted selection [60]. In practice, breeding programs are now underway to combine genetic traits that increase boron uptake and plant access to boron in the soil with *B. napus* cultivars that are efficient in boron utilization [19,29]. Boron efficiency has been assessed using high-throughput phenotyping systems, which facilitate the identification of tolerant cultivars [29].

Under B-deficient conditions, elemental analyses indicate that B-efficient cultivars accumulate equal amounts of B in their tissues as do their inefficient counterparts. Moreover, B-efficient genotypes are characterized by greater B use efficiency [13]. Conversely, boron-inefficient cultivars tend to have accumulated B also as a result of failing to properly metabolize it, which manifests as a disrupted nutrient balance and severe symptoms of deficiency. Boron deficiency destabilizes the vasculature, causing blockages in carbohydrate transport. Soluble sugars in the cotyledons of B-deficient plants have been shown to increase significantly [29]. This accumulation indicates impaired phloem loading and transport and may lead to feedback inhibition of photosynthesis. B-efficient cultivars, however, seem to assimilate sugars into biomass more effectively, alleviating the deleterious effects of carbohydrate accumulation on total plant growth [29]. Root System Architecture (RSA) adaptation to nutrient deficiencies is a well-known phenomenon [82]. Sensitive cultivars develop fewer and thinner adventitious roots and lateral roots under B deficiency conditions, resulting in lower total root mass. In B-efficient cultivars, this modification is less extreme, and a stable RSA may be important for enhancing uptake and ensuring growth under low B availability. Comparative studies on deficiencies of other nutrients (i.e., Ca, N, P, K, and Fe) have highlighted the specificity of the B deficiency response, with tolerance being associated with B-specific traits in efficient genotypes [29].

## 4. Implications of Boron Deficiency Tolerance in Rapeseed

Boron deficiency-tolerant rapeseed genotypes can be developed and utilized for their agronomic, economic, and environmental benefits. Identifying B-efficient rapeseed genotypes and concentrating on their cultivation can reduce the yield loss related to B deficiency. Several genotypes with a high tolerance to B-deficient settings have previously been characterized [29]. These genotypes possess an enhanced B efficiency, enabling them to accommodate the restriction of B availability [29,50]. Furthermore, the mutations in genes, such as *BnaA3* polymorphisms in NIP5;1, which encodes a boric acid channel, have been associated with enhanced low-B tolerance [28,50,61], highlighting potential targets for breeding. The creation of B-efficient rapeseed cultivars will economically lead to increased seed yield, and decrease dependence on costly B fertilizers which have mainly short-term effects [1]. The B-efficient genotypes may save nitrogen input and increase yield by more than 40% under B-deficient conditions, and can gain economic benefits [36]. The use of B-efficient genotypes is an environmentally friendly approach to providing B to crops as it minimizes the risk of chemical fertilizer application which leads to environmental pollution [1]. In addition, such genotypes can assist in sustaining soil health by preventing excessive applications of B fertilizers, which may cause toxicity problems in some crops [1,73,83].

### 4.1. Enhanced Crop Resilience and Yield Stability

The finding of genetic resources for B-efficient rapeseed varieties would assist in breeding to alleviate the problems of B deficiency, since B deficiency is a universal constraint on rapeseed performance. Such cultivars are vital for achieving stable yields in the face of variable field conditions because they are capable of sustaining healthy growth and reproductive functions in soils where boron becomes limited. This robustness minimizes the threat of large-scale yield failures, thus offering farmers better assurance of their crops and supporting food security and a steady supply of oilseeds [28,61,75,84]. This combines QTL mapping and transcriptomic analysis, helping to discover candidate genes that control the boron efficiency of rapeseed. Various studies have proven functional variants and some of them are directly linked to the soil environmental interaction; some helpful alleles were confirmed (e.g., a nodulin 26-like intrinsic protein gene was recognized as a major determinant of boron uptake and allows the plant to thrive in boron-deficient soils) [28,61]. This information regarding genetic traits is very useful in programs for breeding rape varieties with greater yield potential and tolerance to nutrient deficiency [28,61,84]. Moreover, breeding for B-efficient rapeseed variety is one of the key components of sustainable crop production systems, which not only enhance more resilient crops for diverse stress environments but guarantee the supply of crucial agricultural produce. Not only does this approach assist farmers in reducing the risk of yield losses due to abiotic stresses, but it also helps in guaranteeing a steady supply of vegetable oil and biofuel, boosting global food security [20,28,61,75] (Table 3).

### 4.2. Reduced Reliance on Chemical Amendments

Rapeseed cultivars exhibit a novel property of increased boron efficiency in utilization and require lower boron levels for growth, which makes them less dependent on boron fertilizers [29]. Furthermore, genetic variations like the *BnaA3.NIP5;1* gene and other relevant genes contribute to increased boron efficiency and would help with the increase of boron-efficient rapeseed variety [50]. This, in turn, cuts production costs and reduces the environmental impact of fertilizer manufacturing and application. This, in turn, limits the risk of boron fertilizers running off, thereby preventing contamination of neighboring ecosystems and corresponding environmental pollution [1]. Moreover, utilizing such genotypes with better boron efficiency can augment seed yield and nitrogen use efficiency, which can offer sustainable ways of nutrient management in agriculture [36].

### 4.3. Integration of Molecular and Agronomic Innovations

Molecular biology has made great progress in elucidating the genes and regulatory cascades involved in boron uptake and transport in plants. Important genes for boron transport including BOR1 and NIP5;1 have been characterized, where BOR1 is a borate exporter and NIP5;1 enables (boric) acid diffusion through membranes [44,91]. These transporters are regulated according to boron availability and contribute to the maintenance of favorable boron homeostasis in plant tissues [46]. The discovery of these transporters has further transformed the concept of boron transport from a passive one to an active one, which is tightly controlled by plants [6,45,46,48]. Integrating molecular insights with traditional breeding programs can facilitate the optimization of nutrient-efficient crop cultivars. The modulation of BOR1 expression has been confirmed to improve plant tolerance at different boron concentrations, which is essential for maintaining fertility and seed yield in boron-limiting environments [62]. Moreover, boron transporter studies in alternative plant species, including poplar and wheat, have contextualized their function underlying stress adaptations and nutrient uptake, which further reinforce putative improvements based on genetic differences in crop performance [92,93]. This merger of molecular biology and traditional breeding complements the global trend toward precision agriculture. Therefore, combining genetic progress with appropriate agronomic practices will be crucial to maximize resource utilization and improve crop performance. Analyzing the regulatory mechanisms underlying boron transporters enables the design of crop genotypes adapted to soils with suboptimal levels of boron, thus reducing the environmental burden of external nutrients [46]. This technique supports higher yield and better quality of crops and promotes sustainable agriculture.

### 4.4. Sustainable Agricultural Practices

The relationship of boron with other nutrients like nitrogen also plays a vital role in determining rapeseed and canola growth and yield. B-efficient cultivars are already proving useful in terms of improved nitrogen use efficiency and seed yield under B-deficient conditions, thereby illustrating the role of nutrient synergy in B-deficiency management [36]. Harnessing this synergistic effect within the coproduction of food and energy may enable high crop productivity with minimal B input. Boron-efficient and -tolerant rapeseed varieties would be a sustainable solution to the agriculture issues caused by boron deficiency. Through these cultivars, half of the nutrient use efficiency and conservation of natural resources are made, helping to maintain the productivity of arable lands in the future [1]. It has also been proposed that the application of a beneficial bacterium species, *Bacillus pumilus*, could increase boron uptake in boron-deficient soils [40], but such treatments must be carefully monitored since the bacterium at high concentrations was reported to have an inhibitory effect on plant growth.

### 4.5. Economic and Market Benefits

Boron-tolerant varieties can substantially increase the economic potential of rapeseed cultivation. Improving yield stability and reducing input costs in boron-tolerant varieties will also lead to a significant improvement in farmers’ profitability. Additionally, the identification and use of boron-efficient *B. napus* cultivars have shown promise in mitigating yield loss in boron-depleted environments [29]. However, these cultivars have shown more efficient use of boron, thus, they do not need much boron to grow successfully. Additionally, boron-tolerant varieties would ensure stability and reliability in the market and ensure an uninterrupted supply chain [94]. In addition, understanding the genetic and physiological mechanisms of tolerance, including the identification of specific marker genes, also plays a role in the development of boron-tolerant varieties. *NIP5;1* facilitates boron uptake and enhances low-boron tolerance in rapeseed [50]. Furthermore, improved quality and stable yields of boron-tolerant varieties can reinforce the competitiveness of rapeseed, a crucial oil crop in the world. The development of these new varieties will allow for the effective cultivation of rapeseed on a range of soil types, from boron-deficient to non-deficient, thus facilitating production at high standards and supply increases to meet the growing global demand for rapeseed [1,27]. Furthermore, it is conducive to the economic sustainability of rapeseed farming and increases its significance in the global markets of oilseed.

## 5. Conclusions and Future Remarks

Boron is vital for plant growth, influencing cell wall integrity, reproductive development, and overall yield, yet both deficiency and toxicity pose significant agricultural challenges. Advances in molecular breeding, marker-assisted selection, and gene editing, such as CRISPR/Cas9, have enabled the identification and modification of key boron transport genes like BOR and NIP family transporters, facilitating the development of boron-efficient cultivars. However, while genetic improvements can enhance boron uptake and utilization, they should be integrated with optimized fertilization strategies, including precision soil testing and controlled-release fertilizers, to ensure efficient nutrient use while minimizing environmental impact. Rather than advocating for the complete elimination of boron fertilization, the goal is to reduce dependency through genetic innovations while maintaining yield and soil health. Future research should focus on validating boron-efficient cultivars under field conditions, further elucidating boron transport mechanisms, and refining sustainable management practices to balance productivity with ecological responsibility.

## Figures and Tables

**Table 1:** Visible symptoms of boron deficiency in rapeseed (*Brassica napus*). The table highlights key morphological and physiological changes, including stunted plant growth, necrosis of root tips, thickened and brittle stems, deformed and chlorotic leaves, impaired flower development, reduced pod formation, and low seed set. These symptoms result from disrupted cell wall formation, impaired nutrient transport, and reduced reproductive success under boron-deficient conditions.

Scientific Name	Boron Concentration	Effects	Duration	References
*Brassica napus* L.	0.25 to 1000 µM	Severe visible symptoms on leaves, root growth inhibition	4 weeks	[30,31]
*Brassica napus* L.	0.25 and 0.10 µM	Deformed morphology, lower viability, easily ruptured cell walls	-	[27]
*Brassica napus* L.	2.5 and 25 µM	Alleviation of Al-induced root growth inhibition	-	[30]
*Brassica napus* L.	-	Increased root pectin content, decreased cellulose and hemicellulose under Cd stress	-	[32,33]
*Brassica napus* L.	Low B	Growth arrest, cell death, changes in cell wall pore size, oxidative burst	-	[3]
*Brassica napus* L.	High B	Increased seed yield, improved seed dry matter accumulation	Field plot trial	[34]
*Brassica napus* L.	Low B	Deformed cell morphology, lower viability, ruptured cell walls	-	[27]
*Brassica napus* L.	7.5 kg/hm^2^	Increased plant height, branch number, kernels per plant	3 years	[35]
*Brassica napus* L.	Low B	Reduced seed yield, lower nitrogen use efficiency	2 years	[36]
*Brassica napus* L.	2 kg ha^−1^	Improved seed yield, higher oil quality, lower erucic acid and glucosinolate contents	2-year field study	[34,37]
*Brassica napus* L.	1.5 kg B/ha	Increased seed and stover yields, improved oil content by 35.6%	3 consecutive rabi seasons	[38]
*Brassica napus* L.	200–800 g B ha^−1^	Increased oil content in seeds by 3.96%	-	[39]
*Brassica napus* L.	-	Enhanced B uptake, inhibited growth under B supply	6 weeks	[40]

**Table 2:** Genes involved in boron uptake and transport across different plant species, including rapeseed (*Brassica napus*), rice (*Oryza sativa*), and *Arabidopsis thaliana*. The table lists key genes responsible for passive and active boron absorption, transport within plant tissues, and boron distribution to reproductive organs. Examples include BOR1 and NIP5;1 in *Arabidopsis*, which regulate boron efflux and influx, respectively, and their homologs in other species that contribute to boron homeostasis and plant tolerance to boron deficiency.

Plant Name	Scientific Name	Gene	Functions	Effects	References
Rapeseed	*Brassica napus*	*BnaA3.NIP5;1*	Encodes a boric acid channel, crucial for B uptake and root growth	Improves low-B tolerance, enhances seed yield	[50,67]
Rapeseed	*Brassica napus*	*BnaA2.NIP5;1*	Essential for B uptake, expressed in root epidermis	Facilitates B translocation to shoots, supports normal growth	[50,67]
Rapeseed	*Brassica napus*	*BnaA2.HKT1*	Functions as a Na^+^ transporter, involved in root xylem Na^+^ unloading	Enhances salt tolerance, supports growth under B deficiency and salinity	[68]
Rapeseed	*Brassica napus*	*BnaA02.NIP6;1a*	Boron transporter, localized in plasma membrane and cytoplasm	Required for plant development, prevents sterility under B deficiency	[53]
Rice	*Oryza sativa* L.	*OsPIP2;4, OsPIP2;7*	Involved in B transport and tolerance	Increased B tolerance via efflux of excess B from roots and shoots	[69]
Rice	*Oryza sativa* L.	*OsNIP3;1*	Boric acid channel, regulates B distribution	Essential for growth under B-deficient conditions	[70]
Rice	*Oryza sativa* L.	*Os04g0477300*	Suppression improves B toxicity tolerance	Tolerance to B toxicity by abolishing transcript function	[71]
Rice	*Oryza sativa* L.	*Fe-SOD*	Antioxidative enzyme activity	Associated with B tolerance through increased expression	[72]
Rice	*Oryza sativa* L.	*BOR1-like genes*	Efflux-type B transporters	Correlated with B deficiency tolerance	[73]
*Arabidopsis*	*Arabidopsis thaliana*	*AtWRKY47*	Regulates plant tolerance to boron toxicity by controlling B concentration in shoots.	Enhanced tolerance to B toxicity with better growth parameters.	[65]
*Arabidopsis*	*Arabidopsis thaliana*	*LBT*	Controls low-boron tolerance, independent of B uptake or transport.	Improved growth under B deficiency; controlled by a monogenic recessive gene.	[74]
*Arabidopsis*	*Arabidopsis thaliana*	*STOP1*	Activates NIP5;1 expression to enhance B uptake by roots.	Increased tolerance to low-B stress and improved growth.	[75]
*Arabidopsis*	*Arabidopsis thaliana*	*NIP5;1*	Boric acid channel for efficient B uptake.	Improved root elongation and fertility under B-limiting conditions.	[76]
*Arabidopsis*	*Arabidopsis thaliana*	*SHB1/HY1*	Increases BOR4 expression to maintain boron homeostasis.	Alleviates excess boron stress and promotes root growth.	[77]
*Arabidopsis*	*Arabidopsis thaliana*	*BOR4*	Efflux-type B transporter for high-B tolerance.	Upregulated under high B conditions, confers tolerance to high B.	[48]
*Arabidopsis*	*Arabidopsis thaliana*	*AtTIP5;1*	Involved in B transport via vacuolar compartmentation.	Increased tolerance to high B toxicity with improved growth.	[78]
*Arabidopsis*	*Arabidopsis thaliana*	LBT	Controls low-boron tolerance, independent of B uptake or transport.	Improved growth under B deficiency; controlled by a monogenic recessive gene.	[74]

**Table 3:** Various amendments and approaches to enhance boron tolerance in rapeseed. The table outlines strategies such as soil and foliar application of boron fertilizers (e.g., boric acid and borax), breeding and selection of boron-efficient genotypes, use of bio-fertilizers to improve nutrient availability, and management practices like optimizing soil pH and moisture levels. Additionally, genetic approaches, including the upregulation of boron transporter genes, are discussed to improve boron uptake, transport, and distribution, ultimately enhancing plant resilience and yield under boron-deficient conditions.

Scientific Name	Technique Name	Concentration	Effect	References
*Brassica napus*	Hydroponic	0.1 µM B	Si improved growth by 34% in shoots and 49% in roots; increased B transporter expression	[85]
*Brassica napus* L.	Solution culture	0.025, 0.5, and 5.0 µg B/mL	Si increased dry matter yield under B deficiency; enhanced B uptake and accumulation	[86]
*Brassica napus*	Not specified	-	Si increased the range between critical deficiency and toxicity concentration for B	[87]
*Brassica napus*	Co-application of N and B	4.5 and 9 kg borax ha^−1^; 180 kg N ha^−1^	Improved N uptake, NUE, seed yield, and N remobilization; yield increased by >40% under B deficiency	[36]
*Brassica napus*	Foliar application of B	0.25% B	Highest growth and yield of rapeseed under no-tilled and rainfed conditions	[88]
*Brassica napus*	Silicon application	0.1 µM B	Improved growth by 34% in shoots and 49% in roots under B deficiency; increased expression of B transporters	[85]
*Brassica napus*	Balanced B and P application	4.5, 9, and 18 kg Na_2_B_4_O_7_·5H_2_O ha^−1^	Enhanced seed yield and PUE; greater soil bacterial diversity with balanced B and P nutrition	[89]
*Brassica napus*	Sulfur and B fertilization	1.5 kg B/ha	Highest seed and stover yields; improved oil and protein content; enhanced nutrient use efficiencies	[38]
*Brassica napus*	Transgenic lines	-	Improved low-B tolerance and seed yield through increased expression of BnaA3.NIP5;1	[50]
*Brassica napus*	RNAi	-	*BnaA3.NIP5;1* promotes root elongation under low-B conditions, important for seed production	[50]
*Brassica napus*	QTL fine mapping	-	Identification of a nodulin 26-like intrinsic protein gene regulating B efficiency	[28]
*Brassica napus*	Transcriptomics-assisted QTL-seq	-	Expedites identification of quantitative trait genes for B-deficiency response	[28]
*Arabidopsis thaliana*	Overexpression	-	Enhanced expression of NIP5;1 improves root elongation under B-limiting conditions	[76]
*Brassica napus* L.	Pectin-mediated cell wall analysis	0.25 and 0.10 μM B	Low-B-tolerant genotype ‘QY10’ showed less cell wall deformation and higher viability compared to ‘W10’.	[27]
*Brassica napus*	Gene expression analysis	0.25 and 0.10 μM B	‘W10’ exhibited higher pectin concentrations and mRNA abundances of pectin biosynthesis-related genes.	[27]
*Brassica napus*	Soil substrate-based cultivation system	Below 0.1 mg B (kg soil)^−1^	Identification of B-deficiency-tolerant cultivars CR2267, CR2280, and CR2285	[29]
*Brassica napus*	Genetic variation analysis	-	Improved low-B tolerance through BnaA3.NIP5;1 gene expression	[67]
*Brassica napus*	Pectin-mediated cell wall analysis	0.25 and 0.10 μM B	Differential tolerance due to pectin-endowed cell wall properties	[27]
*Brassica napus*	Alternative splicing analysis	-	Increased transcriptome diversity and tolerance in B-efficient cultivar QY10	[90]
*Brassica napus*	Co-application of N and B	4.5 and 9 kg borax ha^−1^	Synergistic effect on seed yield and nitrogen use efficiency	[36]
*Brassica napus*	Transcriptomics-assisted QTL mapping	-	Identification of nodulin 26-like intrinsic protein gene for B efficiency	[28]
